# Mitochondrial Neurogastrointestinal Encephalopathy (MNGIE) Disease

**DOI:** 10.34172/aim.2022.132

**Published:** 2022-12-01

**Authors:** Reza Shervin Badv, Masood Ghahvechi Akbari, Morteza Heidari, Moeinadin Safavi

**Affiliations:** ^1^Pediatrics Neurology Department, Children’s Medical Center, Tehran University of Medical Sciences, Tehran, Iran; ^2^Physical Medicine and Rehabilitation Department, Children’s Medical Center, Tehran University of Medical Sciences, Tehran, Iran; ^3^Molecular Pathology and Cytogenetics Division, Pathology Department, Children’s Medical Center, Tehran University of Medical Sciences, Tehran, Iran

**Figure 1:**
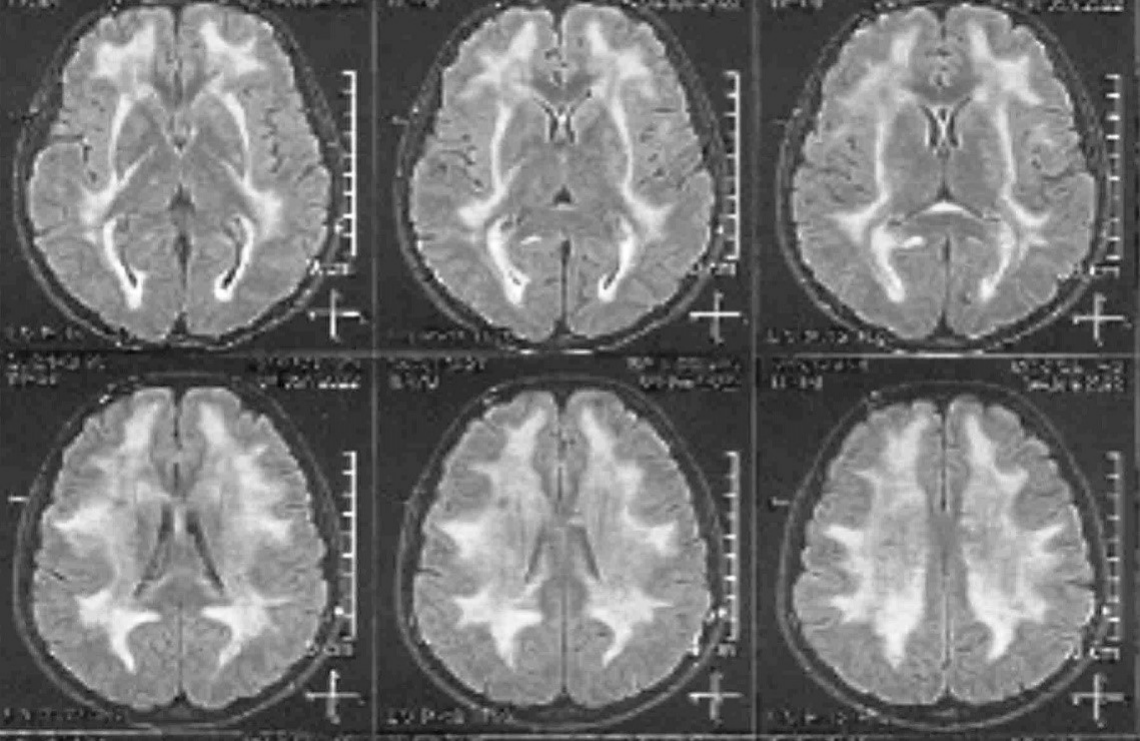
Axial view of brain MRI in FLAIR sequences revealed a prominent leukodystrophy with U fiber sparing

 A 25-year-old male patient and the third child of closely related parents (double first cousins) complained of lower extremity weakness and loss of sensation in the lower extremities. He was hospitalized several times due to gastrointestinal problems. The last time, he was hospitalized due to stomach distention without obstruction (pseudo-obstruction). The patient was apparently cachectic, and had slight ptosis. He had a history of severe tooth decay that was treated by implant surgery. He had severe myopia and was mildly hard of hearing.

 On neurological examination, there was a decrease in sensation and force of the lower limb. Therefore, nerve conduction velocity (NCV) test was performed that showed severe demyelinating sensory motor polyneuropathy with axonal damage in the lower limb. Brain magnetic resonance imaging (MRI) demonstrated a diffuse hyperintensity of white matter in T2-weighted and FLAIR sequences that was in favor of a leukodystrophy with sparing of U fibers (Figure 1). Finally, whole exome sequencing was requested that showed a homozygous pathogenic variant in thymidine phosphorylase (*TYMP*) gene as c.866A > C (p.Glu289Ala).

 MNGIE disease (OMIM# 603041) is a rare mitochondrial disease with autosomal recessive inheritance caused by mutations in a nuclear gene, thymidine phosphorylase (*TYMP*) (OMIM* 131222), and is characterized by progressive gastrointestinal (GI) manifestations (like dysphagia, gastroesophageal reflux (GERD), nausea, postprandial vomiting, gastroparesis, intestinal pseudo-obstruction, episodic abdominal pain and/or distention) due to GI dysmotility, cachexia, ptosis and/or ophthalmoparesis, leukoencephalopathy, and sensory neuropathy (presenting as paresthesia) as well as motor neuropathy (causing bilateral and distal weakness that affect lower extremities more prominently) due to demyelination of peripheral nerves. The order of manifestations is not predictable but the gastrointestinal symptoms are more prominent than neurologic ones. MNGIE disease usually begins between the first and fifth decades of life and in about 60% of patients, manifestations commence before the age of 20 years.^1,2^

 Treatment of MNGIE disease is usually supportive and include antibiotics therapy for abdominal cramps due to intestinal bacterial overgrowth (if proven by hydrogen breath test), stool softeners (as long as there is no diarrhea) and prokinetic drugs like domperidone for GI dysmotility, improving nutritional status by appropriate vitamins and supplements like vitamin D, iron, magnesium, and coenzyme Q for cachexia. Allogenic hematopoietic stem cell transplant has also been proposed for the treatment of these patients.^3^ Dialysis to eliminate toxic metabolites, orthotopic liver transplantation, and enzyme replacement therapy are also considered as alternative treatments.^4-8^
